# An Error in Allen’s Bonninghausen

**Published:** 1897-04

**Authors:** A. McNeil

**Affiliations:** 784 Van Ness Avenue, San Francisco


### AN ERROR IN ALLEN'S BONNINGHAUSEN.

Editor of The Homeopathic Physician:—Permit me to call the attention of your readers to an error of arrangement, of great importance, in Allen's edition of Bönninghausen's *Therapeutic Pocket-Book.*

On page 225 is the rubric: "Spots of Petechiae," and then below it come several other rubrics in the following order: Red, Bluish, Brownish, Coppery, Fiery, Pale, Becoming Pale in the cold. This last, as a reference to the Guiding Symptoms and as the Encyclopedias shows, should read: "Becoming *red* in the cold." After this rubric the next one is "violet," next "like red wine." All arranged as if varieties of petechiae. Now in all these rubrics "Spots" is the word implied, not "Spots of Petechiae" as Allen gives it.

Fraternally yours, A. McNeil.

784 Van Ness Avenue, San Francisco,
March 8th, 1897.

